# Effect of Grain Structure and Ni/Au-UBM Layer on Electromigration-Induced Failure Mechanism in Sn-3.0Ag-0.5Cu Solder Joints

**DOI:** 10.3390/mi13060953

**Published:** 2022-06-16

**Authors:** Yuanxiang Zhang, Jicheng Zhang, Yong Wang, Yike Fang

**Affiliations:** 1 Key Laboratory of Air-Driven Equipment Technology of Zhejiang Province, Quzhou University, Quzhou 324000, China; 2112102426@zjut.edu.cn; 2 College of Mechanical and Electrical Engineering, China Jiliang University, Hangzhou 310018, China; zhangjicheng@cjlu.edu.cn; 3 College of Mechanical Engineering, Zhejiang University of Technology, Hangzhou 310014, China; 4 Chang’an Dublin International College of Transportation, Chang’an University, Xi’an 710064, China; yike.fang@ucdconnect.ie

**Keywords:** electromigration, microstructure, solder bump, failure mechanism, misorientation angle

## Abstract

The development of advanced electronic devices leads to highly miniaturized interconnect circuits (ICs), which significantly increases the electromigration (EM) phenomenon of solder and circuits due to higher current density. The electromigration of solder joints under high current density has become a severe reliability concern in terms of microelectronic product reliability. The microstructure of the solder plays an important role in the electromigration induced degradation. In this study, Sn-3.0Ag-0.5Cu solder bumps with Ni/Au under bump metallization (UBM) layer were fabricated and electromigration acceleration tests were conducted under current density of 1.4 × 10^4^ A/cm^2^ and 120 °C to investigate the effect of grain structure and Ni/Au-UBM layer on EM-induced failure. Grain structures of solder bumps were determined by utilizing the Electron Backscatter Diffraction (EBSD) technique, and single-crystal solder, single-crystal dominated solder, and polycrystalline solder are observed in different test samples. According to the Scanning Electron Microscope (SEM) images, it is observed that the Ni/Au-UBM layer of the Cu pad can inhibit atom diffusion between solder bump and Cu pad, which reduces the consumption of Cu pad but causes a large void and crack at the interface. The EM lifetime of single crystal solder bumps is lower than that of polycrystalline solder bumps when the c-axis of single crystal solder bumps is perpendicular to the electron flow direction. Additionally, the single crystal structure will increase the brittleness of the solder bump, and cracks are easily generated and expanded under the stress caused by the mismatch of thermal expansion coefficients between the solder bump and Ni/Au-UBM layer near Cu pad. Polycrystalline solder bumps with a higher misorientation angle (15–55°) have a higher atom diffusion rate, which will result in the acceleration of the EM-induced failure.

## 1. Introduction

The solder joint current density increases as the electronics industry continues to strive for ever-increasing standards of performance and miniaturization. Electromigration is a mass diffusion process that occurs when momentum is transferred from conducting electrons to diffusing metal atoms at high current densities [1,2]. Consequently, the cathode end of the material gets depleted while the anode end accumulates, increasing resistance over time and possibly leading to device failure. The factors affecting electromigration have been studied and it is believed that current density, temperature, and solder joint composition had a significant influence on electromigration [3,4,5]. In addition, the microstructure of the solder joint also has a significant effect on the electromigration which has attracted many researchers’ attention [6,7,8,9,10,11,12,13,14,15,16,17,18,19].

Sn grains are the matrix of main-stream lead-free solders, and the EM-induced damage will highly depend on the Sn orientation. Lu et al. [6] proposed two EM degradation mechanisms in high Sn-based Pb-free solder joints that are associated with different diffusion processes. Mode-I is associated with Sn self-diffusion when the c-axis is perpendicular or completely misaligned with the current direction, which results in a void formation along the cathode interface. Mode-II occurs as the Sn c-axis was aligned with the current direction, which results in fast dissolution of UBM along the crystal c axis. Lee et al. [8] also reported that the β-Sn grain orientation played a crucial role in the dissolution rate of the cathode Cu, which resulted in different EM failure lifetime. Ho et al. [11] reported that the highly anisotropic electromigration behavior of Cu in the Sn lattice would govern the Intermetallic Compounds (IMC) growth and the Cu depletion, which could seriously threaten the reliability of solder joints. Tian et al. [12] investigated the effects of β-Sn c-axis on the behavior of electromigration in Sn-3.0Ag-0.5Cu solder and found that the growth direction of Cu_6_Sn_5_ IMC in solder matrix will, along the c-axis, accompany growing into solder matrix or gather at the surface of the cross section. Ni et al. [13] reported that self-diffusion was the dominant degradation mechanism and a strong effect of the grain orientation in the Sn-3.0Ag-0.5Cu solder on the time to failure.

From the above published works, Sn grain orientation can influence atomic diffusivity and the growth of IMC during EM process. The brittle interface IMC may reduce the reliability of the solder joints, while the thin and continuous IMC can effectively improve the reliability of lead-free solder joints [15,16]. As significant atom diffusion rate will lead IMC grow faster, the efficient way to inhibit the growth of IMC is the use of Ni/Au UBM layer, which can suppress Cu diffusion as a barrier and its diffusion rate is slower than that of Cu [17,18,19], hence both grain structure of solder and Ni/Au UBM layer would have a great influence on atomic diffusivity during EM process. However, how it can affect the reliability of solder joint under electromigration when considering both grain structure and Ni/Au UBM layer is still not very clear.

In our previous study [20], the experimental results established the relationship between grain structure and the electromigration failure mode of the solder bump, however, the effect of Ni/Au UBM layer was ignored. In this study, the electromigration acceleration tests were carried out on the Sn-3.0Ag-0.5Cu solder bumps. SEM and EBSD analysis were utilized to examine the microstructure change of the solder bumps and investigate the effect of grain structure and Ni/Au-UBM layer on EM-induced failure mechanism, and the competition between consumption of Cu and crack along the interface was also studied from the perspective of atomic diffusivity. This research work will provide useful information to explore the EM-induced failure mechanism in the solder joints.

## 2. Experimental Procedures

**Figure 1:**
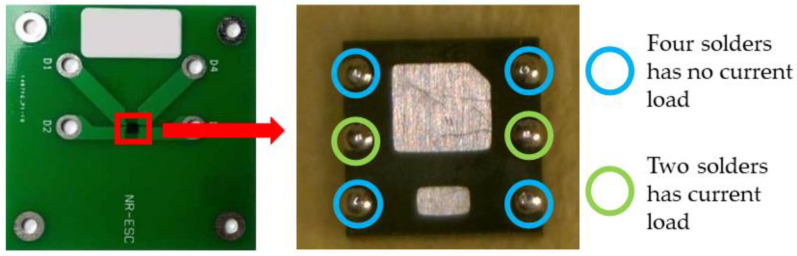
Schematic diagram and inner structure of BGA device.

**Figure 2:**
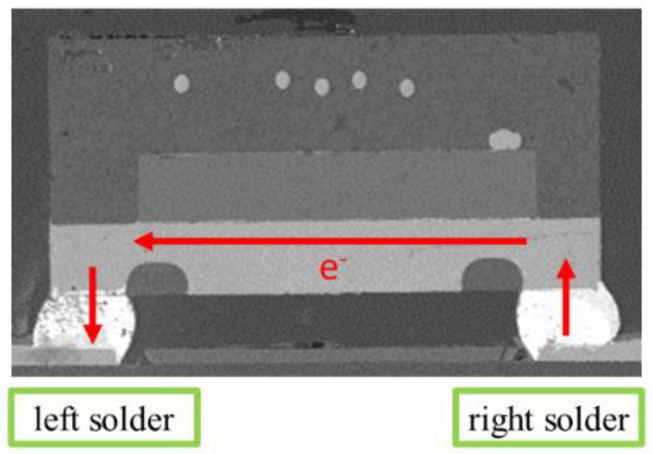
Electron flow direction (opposite current direction) during the EM test.

**Figure 3:**
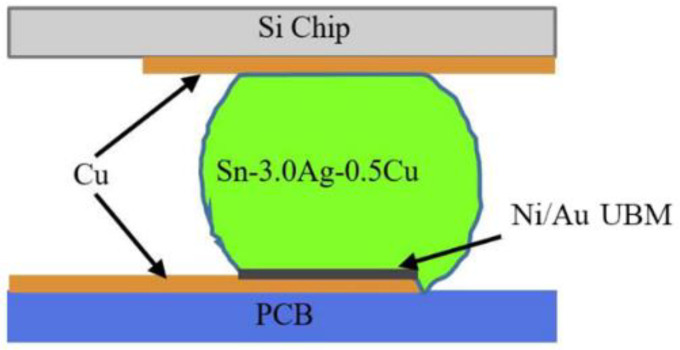
Cross section of solder bump.

Accelerated electromigration test is often used to study electromigration. At normal device operating conditions, the EM lifetime is usually very long, even more than a few years. In order to get results from EM-induced failure in a reasonable amount of time, an accelerated electromigration test was carried out at much higher current densities and elevated temperatures [21]. In this paper, the test samples were fabricated for the electromigration acceleration tests in our previous study [20], as shown in Figure 1. A FDMA8051L chip of Ball Grid Array (BGA) device from ON Semiconductor was bonded on a Printed Circuit Board (PCB) via 6 solder joints. The size of PCB is 50 mm × 50 mm × 1 mm, and the whole BGA structure is only 2 mm × 2 mm × 1.07 mm. The material of the solder is SAC305 (Sn-3.0Ag-0.5Cu) with a diameter of 0.3 mm, and UBM material is Ni/Au. Only two solder bumps located in the middle of the BGA device were subjected to the current for the EM test, and the remaining solder bumps mainly supported the chip on the copper pad of the PCB. During the EM acceleration test, electrons will move from the right to left direction (opposite current direction), passing through the middle two solder bumps and chip, as shown in Figure 2. As shown in Figure 3, the bottom side of the solder has SAC305-Ni/Au-Cu interface because a thin film of Ni/Au UBM layer was coated on the Cu pad, which can inhibit atoms’ diffusion between the solder and pad. In contrast, the upper side of both solders has the SAC305-Cu interface structure because there is no UBM layer on the Cu trace of the chip. This solder bump structure has the advantage of examining the influence of Ni/Au UBM on both scenarios when electrons enter and exit solder bump.

During the EM acceleration test, all test samples were placed in a thermostat and carried a constant current density and temperature. From our previous study [20], solder bumps were subjected to the accelerated EM test at 120 °C (monitored at the top of the chip) with a current density of 1.4 × 10^4^ A/cm^2^ to observe the evolution of microstructure clearly, including the IMC formation, crack extension, and kirkendall void. The 10% increment of resistance was used as a criterion for EM failure [4,5,22]. To better investigate the difference of microstructure evolution among different samples in this study, the test proceeded for about twice of its EM lifetime after increasing the resistance to 10%. SEM and EBSD were used to examine the microstructure of the solder bumps and measure the grain orientation of Sn, which were analyzed to investigate the effect of grain structure and Ni/Au-UBM layer on the electromigration induced failure mode of the solder bump.

## 3. Results and Discussion

Three samples were performed in EM acceleration test, referred to as Sample 1#, Sample 2#, and Sample 3#. As shown in Table 1, Sample 1# demonstrated a short EM lifetime of 43.5 h with EM treatment time of 100 h, while the EM lifetimes of Sample 2# and Sample 3# were very close to each other (86.6 h and 78.5 h separately), and both suffered EM treatment time of 200 h.

**Figure 4:**
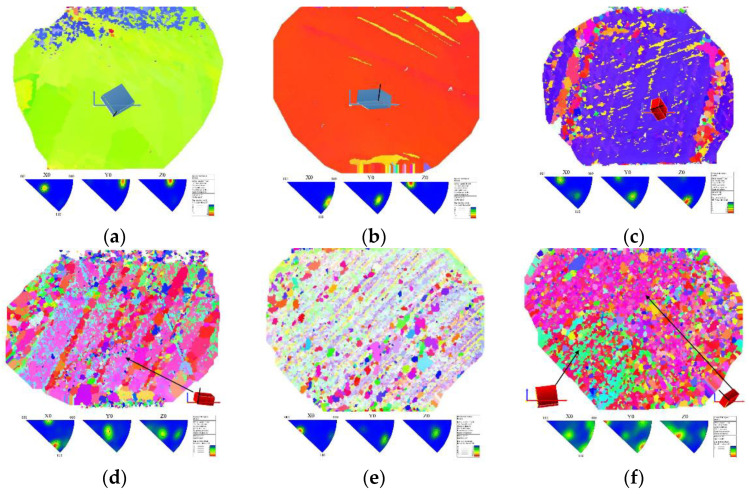
EBSD images with the inverse pole figures of three samples. (**a**) Sample 1#L, (**b**) Sample 1#R, (**c**) Sample 2#L, (**d**) Sample 2#R, (**e**) Sample 3#L, (**f**) Sample 3#R.

Figure 4 shows the EBSD images with the inverse pole figures of three samples for both left and right solder bumps that have gone through EM tests. In Figure 4a, the left solder bump of Sample 1# (Sample 1#L) has a large single grain which has the angle between c-axis of grain and electron is approximately 90°. The left solder bump of Sample 2# (Sample 2#L) can be considered as a single grain that incorporated some band-shaped grains while the angle between c-axis of the grain and electron flow is approximately 75°, as illustrated in Figure 4c. On the contrary, the left solder bump of Sample 3# (Sample 3#L) exhibits a polycrystalline type of structure, consisting of a large number of small grains, as demonstrated in Figure 4e.

It is noteworthy that the right solder bump for Sample 1# (Sample 1#R) also has a large single grain (like the left solder bump), but the angle between the c-axis and the electron flow is only 10°, which is approximately parallel to the electron flow of right solder bump. In addition, polycrystalline grain structure was predominated in right solder bumps of Sample 2# (Sample 2#R) and Sample 3# (Sample 3#R).

**Figure 5:**
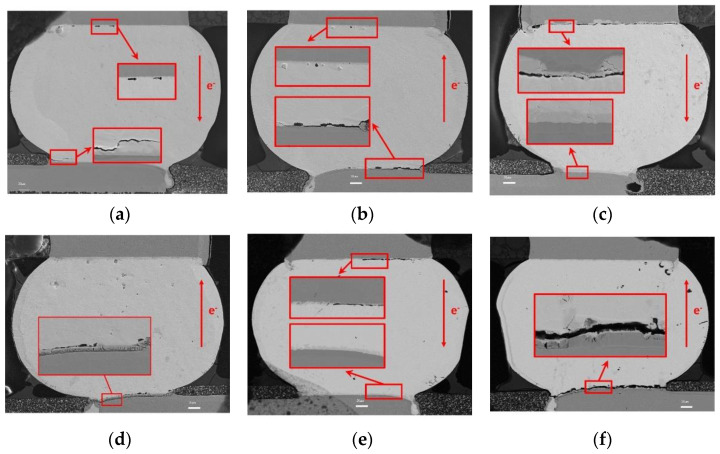
Interfacial failure modes of three samples. (**a**) Sample 1#L, (**b**) Sample 1#R, (**c**) Sample 2#L, (**d**) Sample 2#R, (**e**) Sample 3#L, (**f**) Sample 3#R.

From our previous investigations [20], finite element analysis of electromigration test samples have been performed to explain the relations between grain orientation and EM lifetime based on the atomic density integral method, whereas it did not give the mechanism for how the grain structure could affect the EM induced microstructure evolution. This paper will focus on the relations between grain structure and evolution of microstructure, and the EM failure mechanism affected by grain structure will also be presented. In addition, to better understand the effect of Ni/Au-UBM layer on EM failure and investigate the failure mechanism that refers to polycrystalline solder, the interfacial failure modes of three samples that after EM test were extracted by SEM scanning, as shown in Figure 5.

**Figure 6:**
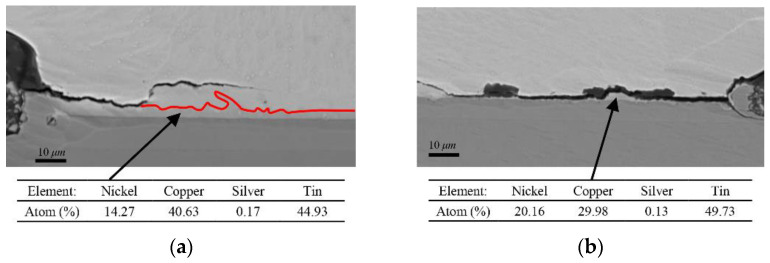
EDX results at the crack area of (**a**) Sample 1#L and (**b**) Sample 1#R.

During electromigration tests, voids usually form in the “current crowding” region where electrons enter the bump from the contacting materials, and then grow and gradually propagate across the interfacial area. As shown in Figure 5, voids or cracks were evident at the cathode end in three test samples, but there was no significant consumption of Cu pad because of the Ni/Au-UBM layer. For Sample 1# in Figure 5a,b, crack extension can be observed near the interface of solder bump and Ni/Au-UBM. It is worth noting that these cracks extend along the interface first and then go through the solder with a sharp crack tip, which shows a significant brittle failure morphology. The Energy Dispersive X-Ray Spectroscopy (EDX) was conducted to investigate element distribution in the microstructure of the crack area, and it showed that the crack initiated at the interface of solder and IMC. For Sample 1#L, the component of IMC should be (Cu, Ni)_6_Sn_5_, and for Sample 1#R, the component of IMC tends to be the (Cu, Ni)Sn, as shown in Figure 6. It indicated that the crack initiated before it formed the stable IMC of (Cu, Ni)_6_Sn_5_, and the mechanical damage dominated the failure of solder.

On the contrary, there were no obvious cracks at the interface of solder bump and Cu pad, and just several small voids were observed along with the interface. For Sample 1#R in EM test, atoms diffusion between solder and Cu pad was inhibited with the existence of Ni/Au-UBM layer on the pad. Atoms of solder at the cathode continuously moved to the anode but no supplement at the cathode weakened the interface of solder bump, as well as Ni/Au-UBM, and then cracks occurred. In addition, Sample 1#L has large tin grain, and its c-axis is almost perpendicular to the electron flow direction. This crystal orientation will accelerate the electromigration-induced degradation according to Lu et al. [6]. Moreover, the single crystal structure increased the brittleness of the solder bump, and cracks were easily generated and expanded under the stress caused by the mismatch of thermal expansion coefficients between the solder bump and Ni/Au-UBM layer near Cu pad. Therefore, Sample 1# has lower EM lifetime compared to Sample 2# and Sample 3#.

For Samples 2# and 3#, as shown in Figure 5c–f, delamination could be found in cathode of all solder bumps, and the cathode consumption was the main cause of the EM failure. The main IMC formed in anode of their left solder bumps was Cu_6_Sn_5_. The IMC thickness in Sample 2#L and Sample 3#L was 8 μm and 4 μm, respectively, as both Samples 2# and 3# suffered the same time of EM treatment, and the cathode of Sample 2#L showed more serious Cu consumption in the Cu trace compared to Sample 3#L. This indicated that Sample 3#L has a lower EM failure process. Meanwhile, the coalescence of large Kirkendall voids lead to a significant band-shaped void, along with the interface in both Sample 2#L and Sample 3#L. Considering sample 2#L has a single crystal dominated grain structure with an angle of 75° approximately between the c-axis and the electron flow, which is beneficial for atom diffusion, it is reasonable to be more prone to electromigration-induced degradation compared to Sample 3#L (with polycrystalline grain structure).

**Figure 7:**
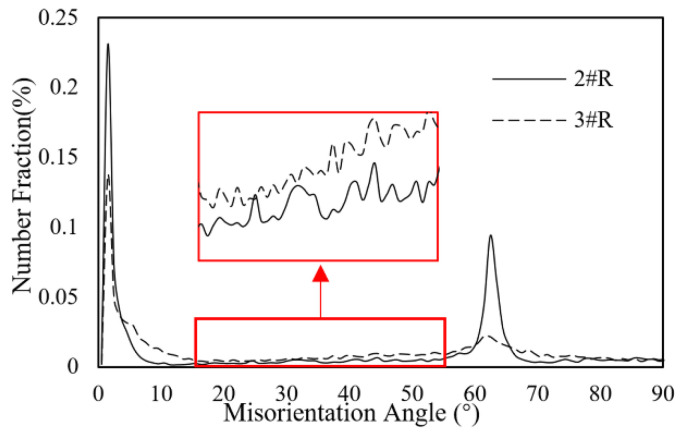
Misorientation angle distributions of Sample 2#R and Sample 3#R.

For Sample 2#R and 3#R, they both had a substantial amount of EM generated void along with the interface of Ni/Au UBM and solder bump in cathode. Especially for Sample 3#R, it had a long gap throughout the interface. Since both the Sample 2#R and 3#R have undergone 200 h electromigration acceleration test, the more serious EM failure of Sample 3#R implied that its internal atomic diffusion speed was much faster than that of Sample 2#R. Sample 2#R and 3#R have a polycrystalline grain structure, and there was no obvious difference in the grain size distribution. The misorientation angles of right solder in Sample 2#R and Sample 3#R were calculated and compared in Figure 7. According to the literature [23], atoms in crystals with the misorientation angle of 15–55° will have a higher diffusion rate. Results indicated that the fraction of misorientation angle in Sample 3#R was larger than Sample 2#R during this range of angle, which was consistent with the difference of microstructure between Sample 3#R and Sample 2#R. Therefore, the misorientation angle will affect the EM lifetime of solder bump significantly for the polycrystalline solder.

## 4. Conclusions

This paper employed electromigration acceleration tests on the Sn-3.0Ag-0.5Cu solder bumps and then utilized SEM, EBSD, and EDX to investigate the effect of grain structure and Ni/Au-UBM layer on EM-induced failure. The main conclusions are as follows:

1. Both Ni/Au-UBM layer and grain structure would affect the EM-induced failure. Voids or cracks are evident at the cathode end in soler bumps during EM, but there is no significant consumption of Cu pad because of the Ni/Au-UBM layer. The presence of Ni/Au-UBM layer on the Cu pad can inhibit atoms diffused between solder bump and Cu pad, and cracks at the interface near Cu pad induced failure that would occur during EM.
2. The EM lifetime of single crystal solder bumps is lower than that of polycrystalline solder bumps when the c-axis of single crystal solder bumps is perpendicular to the electron flow direction. Moreover, the single crystal structure increased the brittleness of the solder bump, and cracks are easily generated and expanded under the stress caused by the mismatch of thermal expansion coefficients between the solder bump and IMC near Cu pad.
3. From the statistical results on the misorientation angle distribution in polycrystalline solder bumps, the electromigration-induced failure will be more likely to occur when the misorientation angles are in the range of 15–55°, which is consistent with the previous literature.

## Figures and Tables

**Table 1:** EM lifetime and EM treatment time for three test samples.

Sample	EM Lifetime/h	EM Treatment Time/h
1#	43.5	100
2#	86.6	200
3#	78.5	200

## Data Availability

Not applicable.
